# Vocalisation Repertoire of Female Bluefin Gurnard (*Chelidonichthys kumu*) in Captivity: Sound Structure, Context and Vocal Activity

**DOI:** 10.1371/journal.pone.0149338

**Published:** 2016-02-18

**Authors:** Craig A. Radford, Shahriman M. Ghazali, John C. Montgomery, Andrew G. Jeffs

**Affiliations:** 1 Leigh Marine Laboratory, University of Auckland, PO Box 349, Warkworth, 0941, New Zealand; 2 Marine Ecosystem Research Centre (EKOMAR), Faculty of Science and Technology, Universiti Kebangsaan Malaysia, Bangii, Malaysia; 3 School of Environmental Sciences and Natural Resources, Faculty of Science and Technology, Universiti Kebangsaan Malaysia, Bangii, Malaysia; Claremont Colleges, UNITED STATES

## Abstract

Fish vocalisation is often a major component of underwater soundscapes. Therefore, interpretation of these soundscapes requires an understanding of the vocalisation characteristics of common soniferous fish species. This study of captive female bluefin gurnard, *Chelidonichthys kumu*, aims to formally characterise their vocalisation sounds and daily pattern of sound production. Four types of sound were produced and characterised, twice as many as previously reported in this species. These sounds fit two aural categories; grunt and growl, the mean peak frequencies for which ranged between 129 to 215 Hz. This species vocalized throughout the 24 hour period at an average rate of (18.5 ± 2.0 sounds fish^-1^ h^-1^) with an increase in vocalization rate at dawn and dusk. Competitive feeding did not elevate vocalisation as has been found in other gurnard species. Bluefin gurnard are common in coastal waters of New Zealand, Australia and Japan and, given their vocalization rate, are likely to be significant contributors to ambient underwater soundscape in these areas.

## Introduction

Many teleost fish produce species-specific sounds using specialised sonic organs. Although the sound production mechanism in fish is not analogous to the laryngeal mechanism in other vertebrates, this behaviour is also commonly termed vocalisation [1,2]. Fish vocalisation is a major contributor to the biotic component of ambient underwater sound in many coastal areas often making a significant contribution to sound energy in the frequency range between 100–1000 Hz [3,4]. However, identification of these soniferous fishes requires ‘sound-truthing’ individual species in isolation since identification in the wild can lead to confusion as to the identity of acoustic source [5]. Given the diversity of fish species and the technical difficulties of sound recording in the wild, sound-truthing is most commonly conducted on captive fish despite the constraints that may impose on natural behaviour. As a consequence, the call repertoire, behavioural context and temporal patterns of vocalisation are less well known in fish than in other vertebrates [6].

Over 800 species of fishes from 109 families worldwide are known to be soniferous [7–9]. In general, the vocal repertoire of a single species is limited to one or two types of sounds, though in some species it may be more extensive [10]. Members of families with extensive vocal repertoires include the toadfishes (Batrachoididae) [11–13], elephantfish (Mormyridae) [14,15], gobies (Gobiidae) [16], damselfishes (Pomacentridae) [17] and gurnards (Triglidaeae)[18–21]. Within a fish species, the extent of a vocal repertoire and the seasonal and daily use of vocalisation may reflect biological function in a way that is useful to the interpretation of acoustic soundscapes. Vocalisations often increase during reproductive seasons and a role for vocalization in territorial defence and/or mate selection has been well documented [8,22–25]. Other agonistic social, and feeding roles have been suggested but are less well known, and the specific role of sound production diversity within a species is still a matter of debate [11–13,26,27].

As part of a temporal and spatial survey of ambient noise at various marine habitats around New Zealand, numerous 24 hour field recordings have been undertaken which include a wide range of fish vocalisations [3,28]. In order to evaluate the acoustic contributions from individual fish species it is necessary to characterise the vocalisation of the most acoustically active species. The bluefin gurnard, *Chelidonichthys kumu*, is a member of the family Triglidaeae, which are well known for their vocalisations. It is a commercially important demersal species that is common in many coastal waters with sand or mud seafloor in New Zealand, Australia, Indo-West Pacific, Japan and Korea [29,30]. The aural descriptions for Triglidaeae vocalisation in general are knocks, grunts and growls, which consist of pulsed sounds ranging in duration from 10–3000 ms and with peaks of sound energy between 250 and 600 Hz [8]. A single study on the vocalisation of *C*. *kumu* recorded two types of dull grunts described as ‘gus’ and ‘pons’ with dominant frequencies ranging from 250 to 300 Hz [31]. The sounds in this species are thought to be produced by the contractions of paired intrinsic sonic muscles that occupy the dorso-lateral surface of the swim bladder[31].

The vocalisation activity and behavioural context of sound production for the bluefin gurnard is currently unknown. In other gurnard species, the grey (*Eutrigla gurnadus*), streaked (*Trigloporus lastoviza*), tub (*Trigla lucerna*), red gurnard (*Aspitrigla cuculus*), northern sea robin (*Prionotus carolinus*) and striped sea robin (*Prionotus evolans*) has been reported to produce agonistic vocalisation sounds [8,18, 19,21]. During competitive feeding, the streaked gurnard, northern and striped sea robin only produced one type of sound (described as a growl, squawk and grunt, respectively) as opposed to the grey gurnard which produced three types of sound (knocks, grunts and growl). In addition to the variable size of the sound repertoire among Triglidaeae species, not much is known with regards to their temporal patterns of vocalisation. The vocalisation activity of the grey gurnard was reported to vary daily with photoperiod and feeding activity but not with temperature or season [32]. This lack of knowledge for members of the Triglidaeae is consistent with our general lack of understanding of the daily periodicity of vocalisation in most soniferous fish species. It is most likely that bouts of vocalisation in fish are not randomly distributed in time. Therefore, determining temporal variability in vocalisation is best achieved through continuous sampling, rather than through intermittent sampling designs. By using continuous acoustic sampling, this study aims to describe the vocalisation repertoire and the daily vocalisation pattern of bluefin gurnard in captivity and investigate possible association with feeding.

This information can be used to help determine the temporal pattern of activity in this species, assuming that vocalisation activity could be used as a proxy for the general state of activity. Additionally, this information would be useful in understanding the contribution of this species to sound in the underwater soundscape. A clear identification of the vocal repertoire of individual species and an understanding of their temporal pattern of vocalisation has the potential to be used for passive acoustic surveys of fish populations in the wild. Furthermore, if the behavioural context of sound production in fish species is also known, such as an association with feeding and reproductive behaviour, it would ultimately allow more detailed interpretation of ambient sound recordings [33–37].

## Materials and Methods

### Fish capture and holding

Bluefin gurnards were captured by the authors in Omaha Bay, North Island, New Zealand using bottom long lines in shallow water (<10 m depth) to minimize barotrauma effects, and set for only 30 min to minimize injury and stress. Captured fish were immediately transported in seawater to the Leigh Marine Laboratory where they were housed in a circular polyethylene tank (opaque, diameter1.6 m; depth 0.6 m) with flow-through filtered (200 μm) aerated sea water supply and sand placed on the floor of the tank. The tank was located in a quiet area outdoors to minimise extraneous noise. The fish were held under ambient photoperiod and sea water temperature (18°C). They were allowed to acclimatize to laboratory conditions for six weeks prior to the experiments and were fed sliced pilchards three times a week. Capture methods provided only female fish so a single group comprising three adult female fish with total lengths (TL) measuring 398, 396 and 395 mm respectively were used throughout the experiments. All experiments and fish capture techniques were conducted under the University of Auckland Animal Ethics Committee approval no. AEC727.

### Experimental setup

Two sets of experiments were conducted using the same group of fishes. The experiments were designed to allow the captive fish to behave as naturally as possible by minimizing human contact and could be observed using an underwater video camera. The first experiment (vocalisation activity) involved continuous recording sessions without providing any stimuli (including food) for vocalisations. This experiment was conducted for five non-consecutive days each lasting 24 hours. The second experiment (feeding related vocalisation) involved introducing food to the fish as a stimulus for competitive feeding. This was done by feeding, without exceeding demand, through a feeding tube one piece of food at a time for 15 min at two specified times (0000 and 1200 hrs). Observations using an underwater closed circuit camera with infrared lighting confirmed food consumption during every feeding session. The feeding experiments were also conducted for five non-consecutive days each lasting 24 h. During both the experiments, water flow and aeration was turned off except over duration of one hour between 0900 to1000 hrs. Recordings made during this time were not analysed.

Experiments commenced at the same hour of the day (1700 hrs) and were conducted from 16 November to 12 December 2008. Information for the sunset (2030 hrs) and sunrise (0555 hrs) times were obtained from the sun data provided online by www.gaisma.com. Dusk and dawn time were defined as beginning one hour before and lasting until one hour after astronomical sunset and sunrise times respectively. Light levels were monitored using data collected by the Leigh Marine Laboratory climate station and for the same time periods (e.g. day, dawn and dusk) levels were similar for the entire recording period.

### Recording of fish sound

For each experiment, sound was recorded continuously for 24 h with a hydrophone (High Tech Inc. USA, HTI 960 min) with a sensitivity of -165 dB re 1 V/1 μPa and a flat frequency response from 0.01 to 30 kHz connected to a portable digital audio recorder (Sound Devices 722, Wisconsin,USA). Sound was sampled at 32 kHz sampling rate (16 bit resolution) and passed through a built-in low cut 40 Hz 18 dB octave filter. All time stamped recordings were analysed using RAVEN Pro 1.3 for Windows (Bioacoustics Research Program, Cornell Laboratory of Ornithology, Cornell, NY, U.S.A.). The placement of the hydrophone (suspended centrally at midwater) took into consideration the effect of tank wall resonance frequency and signal attenuation distance [38]. The calculated minimum resonance frequency was 1441 Hz and the attenuation distance, where sound pressure decreased by 20 dB, was 0.46 m.

### Analysis of vocalisation sound and activity

For the characterization of recorded fish sounds, individual sounds were selected from recordings based upon *a posteriori* classification of aurally distinguishable sound types, i.e.; grunts and growls. Only sounds that could be clearly identified aurally and showed a clear visual representation in both oscillogram and spectrogram were considered. Acoustic measurement of the sounds were made after filtering the sound through a digital bandpass filter between 40 and 1200 Hz. The following sound parameters were measured; sound duration, SD (time elapsed between the start of the first pulse to the end of the last pulse measured in ms); pulse duration, PD (mean time elapsed between start and the end of a pulse measured in ms); number of pulses, PN; pulse period, PP (mean time elapsed between the peak amplitude of two consecutive pulses measured in ms); pulse interval, PI (mean time elapsed between the ending and the beginning of two consecutive pulses measured in ms); peak frequency, Pf (the frequency component with the highest amplitude in the entire sound); lower frequency, Lf (lower frequency limit which amplitude is 3 dB less than the peak frequency); upper frequency, Uf (upper frequency limit which amplitude is 3 dB less than the peak frequency), 3 dB bandwidth, 3dB Band Width ((BW) range between lower and upper frequency)) and damping coefficient, Q (Q = Pf/3 dB bandwidth). All temporal parameters were measured from the oscillogram (averaged for all or up to ten pulses in long sounds) while spectral parameters were measured from spectrograms and power spectra calculated using a 750-point Fast Fourier Transform (filter bandwidth of 61.4 Hz) with a Hanning window. The adequacy of the aural classification were validated using linear discriminant analysis (LDA) based on the extracted sound parameters except for the parameter 3dBBW since it was highly correlated with the frequency limit values. Comparisons between the mean sound parameters within each of the two different types of grunts (Gru1 vs. Gru2) and growls (short vs. long) were compared with t-tests and applying a sequential Holm-Bonferroni correction to significance level to control for inflated Type I error arising from multiple comparisons.

For the diel vocalisation experiment, the occurrence of different sound types was counted from dividing the entire sound recording into 10 sec resolution time frames. The sounds were pooled among all individuals since they could not be traced to individual fish from the recording. Vocalisation activity is represented by calculating the vocalisation rate (sounds fish^-1^ day^-1^ or h^-1^) where values for single fish calculated by dividing the overall counts with the total number of fish (*n* = 3). The mean hourly vocalisation rate were compared among different periods of the day (day, dusk, night and dawn) with repeated measures ANOVA (rmANOVA) since the same group of individuals were used throughout the experiment. Following a significant rmANOVA result, the *post hoc* multiple group comparisons were conducted with Tukey HSD tests. Correlation analysis was used to compare the occurrence of grunt and growl vocalisations while the Wilcoxon Signed Ranks Test was used to compare the prevalence of the different type of sound between day and night.

For the feeding experiment, the occurrence of vocalisation was counted in 15 min time periods (sound fish^-1^ 15min^-1^) encompassing the duration of two hours before, during and two hours after feeding commenced. This duration was from 1000 to 1400 hrs for the noon feeding and 2200 to 0200 hrs for the midnight feeding respectively. The Friedman’s test was used to compare the mean amount of vocalisation among the 15 min time periods (*n* = 16) which include periods during feeding session (*n* = 1) and non-feeding sessions (*n* = 15). Association of vocalisation with feeding is assumed when a significant variation in the amount of vocalisation is observed throughout the observation duration (4h) centered at feeding. All statistical analyses were calculated using the software SigmaPlot Ver 11.0.

## Results

### The vocalisation repertoire and acoustic characteristics

**Fig 1:**
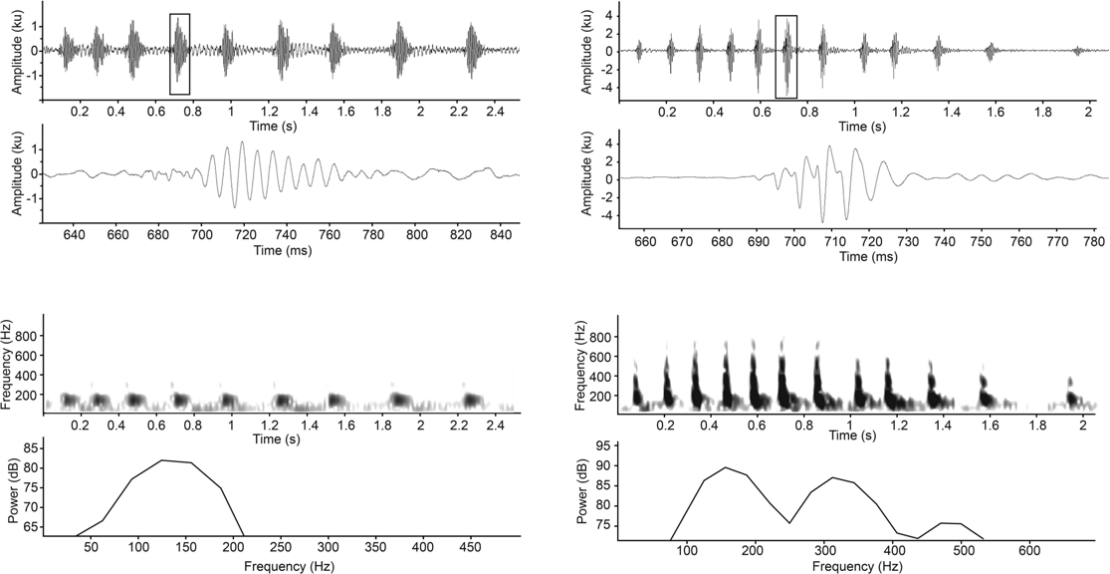
Oscillograms (whole sound and enlarged section), spectrogram and power spectrum (750 point FFT; Hanning window; 64 Hz filter bandwidth) for representative examples of; a) Gru1 sound, b) Gru2 sound.

**Fig 2:**
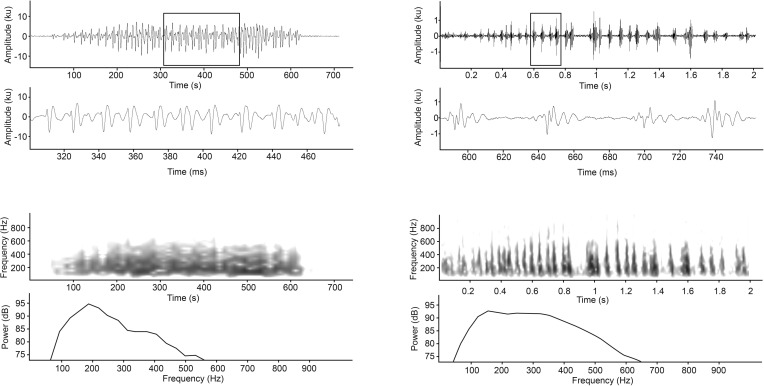
Oscillograms (whole sound and enlarged section), spectrogram and power spectrum (750 point FFT; Hanning window; 64 Hz filter bandwidth) for representative examples of; a) Short-growl sound and, b) Long-growl sound. Amplitude and power are shown as relative units.

Sounds produced by the bluefin gurnard consisted of short and repeated low frequency pulses. Aurally, four different types of sound were deduced from the recordings that could be grouped into two general categories, “grunts” and “growls” (Figs 1 and 2). The grunt consisted of two subtypes, Gru1 and “Gru2” (Fig 1A and 1B). The growl, which is reported for the first time for this species, also consisted of two subtypes, the “Short-growl” and “Long-growl” (Fig 2A and 2B). The adequacy of the different aural categories was supported by LDA of a set of 318 randomly selected pulsed sounds in a train (*n* = 318, Kolmogorov-Smirnoff = 0.23, p<0.01). Single pulsed sounds were excluded from the analyses due to the absence of the variable PP and PI. Approximately 83% of the total sounds were correctly classified by the LDA; Gru1 74.4%, Gru2 83.2%, Short-growl 79.7% and Long-growl 95.2%) (Table 1). The variables PN, PD and PRR were the main contributors to the discriminant functions (Table 2). SD for all sound types were relatively long exceeding 2 s for grunts (Gru2 2.27±0.15 s and Gru1 2.44±0.14 s) and exceeding 1 s for growls (Short-growl 1.66±0.18 s and Long-growl 2.66±0.20 s). The waveform of the pulse was consistent within sound types. Grunts had 3 to 8 wave cycles per pulse, whereas growls had 1 to 3 wave cycles per pulse. Mean PN were similar for Gru1 and Gru2, but were less in number (mean = 11.0± 0.5 pulses) compared to growls (mean = 28.2± 1.3 pulses). Consequently grunts had longer temporal characteristics of the pulses, as measured by PD, PP and PI (Table 3). Grunts had lower Pf1 (129±1.3 Hz and 144±1.4 Hz) than growls (190±5.4 Hz and 215±5.8 Hz) but had a harmonic component (secondary peak frequency, Pf2) at approximately an octave higher than Pf1. It appeared that the harmonic was more pronounced in Pon-grunt with a smaller difference between peak levels (5–10 dB) as compared to a >10 dB difference in Gru1 (Fig 1A and 1B). The growls lack a harmonic component and had broader 3dBBW. The PRR for the Short-growl (26.2±1.5 pulses s^-1^) was twice as fast as the Long-growl (12.3±0.7 pulses s^-1^), and five times faster than the grunts (Gu-grunt 4.8±0.1 pulses s^-1^ and Pon-grunt 5.6±0.2 pulses s^-1^). Q values were relatively low with means of 1.3±0 for growls and 2.0–2.2±0 for grunts.

**Table 1:** Classification of sound types to four categories by linear discriminant analysis.

True Group				
Sound Type	Gru1	Long Growl	Gru2	Short Growl
Gru1	58	0	20	0
Long Growl	0	59	0	12
Gru2	20	0	99	0
Short Growl	0	3	0	47
Total, *n*	78	62	119	59
Number Assigned				
Correct	58	59	99	47
Proportion (%)	74.4	95.2	83.2	79.7

*n* = 318; *n* Correct = 263; Proportion Correct = 82.7%

**Table 2:** Linear discriminant functions for the validation of sound type classification. SD = sound duration; PN = number of pulses; PD = pulse duration; PP = pulse period; PI = pulse interval; PRR = pulse repetition rate; Pf = peak frequency; Lf = lower frequency; Uf = upper frequency 3dBBW = 3 dB bandwidth; Q = damping coefficient.

Discriminant Functions				
Variables	Gru1	Long Growl	Gru2	Short Growl
Constant	-95.56	-94.24	-98.78	-121.37
SD	0	0	0	0
PN	0.46	0.74	0.45	0.96
PD	0.45	0.29	0.32	0.26
PP	-0.01	-0.08	-0.02	-0.08
PI	0.10	0.10	0.11	0.10
PRR	0.48	0.58	0.49	1.02
Pf1	-0.45	-0.41	-0.47	-0.43
Lf1	-0.32	-0.06	-0.33	0.04
Uf1	0.65	0.66	0.69	0.68
Q1	63.58	48.16	68.37	46.74

**Table 3:** Acoustic features measured in Gru1, Gru2, Short-growl and Long-growl sounds. Values are means ± SEM and range is given in parentheses below. For Pf, Lf and Uf the median value is given in parentheses on the right. SD = sound duration; PN = number of pulses; PD = pulse duration; PP = pulse period; PI = pulse interval; PRR = pulse repetition rate; Pf = peak frequency; Lf = lower frequency; Uf = upper frequency 3dBBW = 3 dB bandwidth; Q = damping coefficient. * = significant difference between means within each of the two different types of grunts and growls using t-tests at (P < 0.05) with sequential Holm-Bonferroni correction for Type I error inflation.

	Grunts		Growls	
Parameters	Gru1	Gru2	Short-growl	Long-growl
*n*	78	119	59	62
SD (s)	2.437±0.135 (0.397–8.137)	2.271±0.151 (0.050–11.856)	1.655±0.178* (0.288–6.815)	2.663±0.197* (0.376–8.660)
PN (pulses)	11.0±0.5 (4–29)	11.1±0.6 (1–39)	33.8±2.1 (6–88)	28.2 ±1.3 (7–56)
PD (s)	0.084±0.003* (0.038–0.139)	0.056±0.001* (0.027–0.105)	0.0153±0.000* (0.009–0.035)	0.0323± 0.001* (0.011–0.057)
PP (s)	0.229±0.005 (0.105–0.372)	0.214±0.004 (0.109–0.434)	0.033±0.002* (0.008–0.112)	0.080±0.004* (0.026–0.195)
PI (s)	0.148±0.005 (0.042–0.257)	0.157±0.004 (0.062–0.353)	0.020±0.001* (0.008–0.050)	0.050±0.004* (0.013–0.174)
PRR (pulse s^-1^)	4.8±0.1* (3.1–10.1)	5.6±0.2* (2.8–20.0)	26.2±1.5* (7.7–59.3)	12.3±0.7* (5.0–36.6)
Pf 1 (Hz)	129±1.3* (125) (95–156)	144±1.4* (156) (125–156)	215±5.8* (218) (125–313)	190± 5.4* (187) (125–313)
Lf 1 (Hz)	96±1.5* (98) (52–118)	110±0.9* (109) (76–130)	137±3.2* (131) (94–210)	123±2.2* (121) (82–181)
Uf 1 (Hz)	163±1.0* (164) (143–187)	177±0.9* (176) (153–193)	313±6.0* (320) (192–393)	276±7.0* (263) (190–407)
3dBBW (Hz)	67±0.8 (58–95)	67±0.6 (57–115)	176±5.5* (88–253)	154±6.4* (83–283)
Q1	2.0±0.0* (1.0–2.7)	2.2±0.0* (1.4–2.7)	1.3±0.0* (0.6–2.2)	1.3±0.0* (0.7–1.9)
Pf 2 (Hz)	276± 2.5* (281) (250–313)	290 ±1.8* (281) (250–314)	-	-
Lf 2 (Hz)	234± 2.0* (230) (187–274)	246± 1.9* (249) (187–281)	-	-
Uf 2 (Hz)	313 ±2.1* (311) (281–346)	326± 1.8* (322) (283–365)	-	-
3dBBW 2 (Hz)	79 ±1.1 (55–99)	80± 0.9 (58–125)	-	-
Q2	3.6± 0.1 (2.6–5.1)	3.7± 0.0 (2.5–4.9)	-	-

A majority of sounds were produced in series or trains with initial pulses having low amplitude which gradually increased and reached the maximum level by the fourth or fifth pulse for a short sound, but after the tenth pulse for longer sounds.

### Diel vocalisation activity

In total, 6368 sounds (5464 grunts and 904 growls) were recorded from the group of three female bluefin gurnard over 5 d (i.e., 5 replicate 24 h periods). Temporal analyses were conducted on the two general sound types (grunt and growl) rather than on individual sound type. The bluefin gurnard produced calls with a mean of 424.5 ± 46.3 call fish^-1^ day^-1^ or 18.5 ± 2.0 call fish^-1^ h^-1^ with an average composition of 85.6% grunts (364.3 ±42.5 call fish^-1^ day^-1^ or 15.8± 1.8 call fish^-1^ h^-1^) and 14.4% growls (60.3±11.8 call fish^-1^ day^-1^ or 2.6± 0.5 call fish^-1^ h^-1^) respectively.

**Fig 3:**
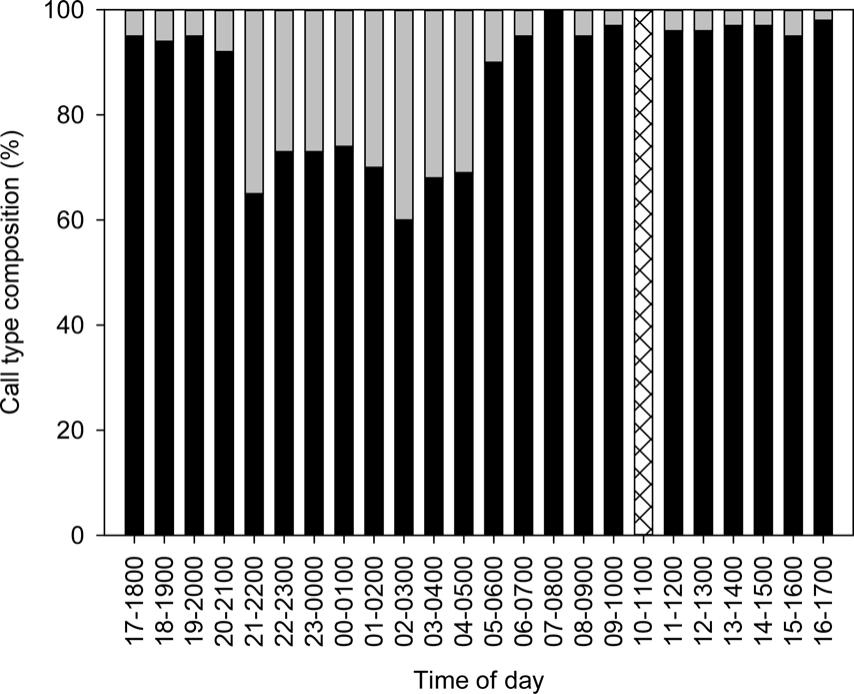
Diel variation in the proportion of grunt and growl type sounds. Grey vertical bar are growls and black vertical bar are grunts. Bar represents mean values observed in the hourly interval over five non-consecutive days of sampling. Crossed bar at 0900–1000 hrs indicate no data due to activation of water flow.

**Fig 4:**
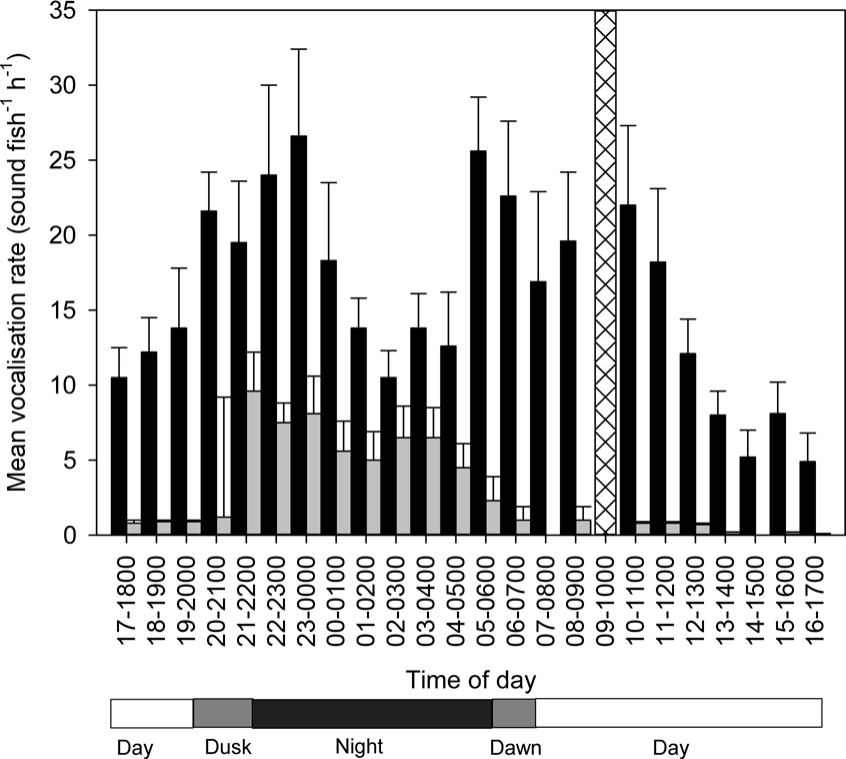
Daily pattern of vocal activity showing mean hourly vocalisation rate (mean ± SEM; *n* = 5). Black bar represents grunt sounds and grey bar represents growl sounds. The single shaded vertical bar at 0900–1000 hrs indicates the activation of water flow.

**Fig 5:**
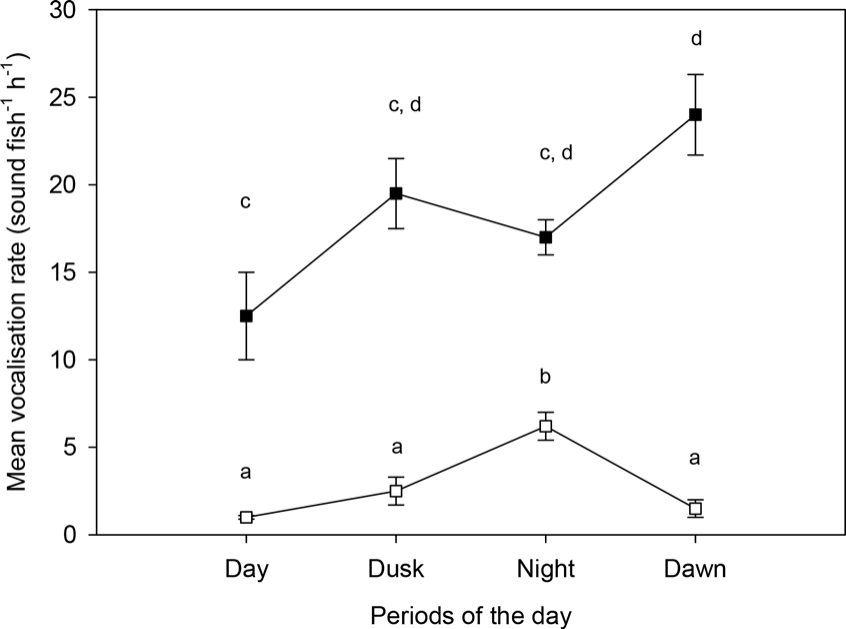
Comparison of vocalisation rate (mean±SEM; *n* = 5) during different periods of the day. Dark square represent grunt sound and white square represent growl sounds. Different letters indicate statistically significant differences between individual means within either growls or grunts (Tukey test; p<0.05).

However, the proportions of the grunts to the growls was significantly different between day and night (p<0.001), primarily due to the large increase in growl vocalisation at night (Figs 3 and 4). Grunts tend to be produced periodically in groups, while growls were produced singly and intermittently. There was no significant correlation between the number of grunt vocalisations and the number of growl vocalisations within an hour (Pearson correlation, r = 0.36; p>0.08) indicating that vocalisation of one call type is independent of the other. There were significant differences in the mean vocalisation rate (i.e., number of sounds fish^-1^ h^-1^) among the different periods of the day for both grunt (rmANOVA F_3_ = 7.44, p = 0.004) and growl vocalisation (rmANOVA F_3_ = 20.50, p<0.001). Tukey tests showed that the number of grunt vocalisation was significantly higher (p = 0.003) at dawn compared to during the day, but not significantly different than other periods, i.e., night and dusk. There were no significant differences in the number of grunt vocalisations among other periods (day, night and dusk). For growls, the number of calls was significantly higher (p<0.005) at night compared to other periods, i.e., day, dusk and dawn. (Fig 5).

Occasional observation using closed circuit television camera showed that sounds were produced in a non-specific behavioural manner whilst individual fish were either swimming or lying on the sandy tank floor. However, strict validation on the context of sound production could not be verified in this study.

### Feeding related sound

**Fig 6:**
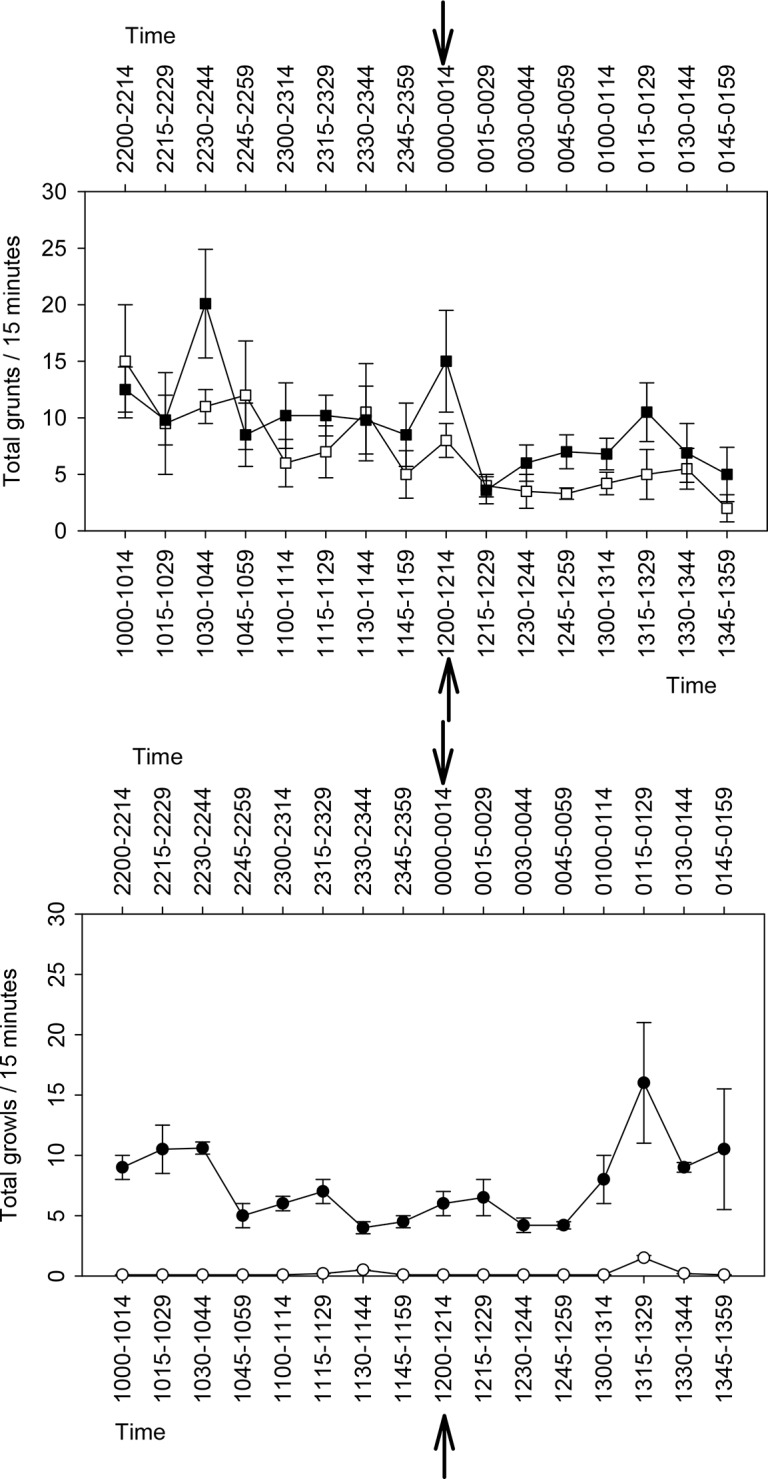
Total vocalisation counted in consecutive 15 min periods from 2 h prior to feeding, during feeding and 2 h after feeding commenced. 5a) Grunts and 5b) Growls. Counts of vocalisations were conducted for 5 non-consecutive days. Solid line and symbol = midnight; dotted line and hollow symbol = noon feeding. Vertical arrow indicates time of feeding. Data represent the mean values ± SEM.

Vocalisation rates of the bluefin gurnard were similar between feeding and non-feeding events for both the grunt and growl call types (Grunt noon χ^2^_15_ = 18.7; p = 0.23; Grunt midnight χ^2^_15_ = 19.8; p = 0.18; Growl noon χ^2^_15_ = 17.3; p = 0.30; Growl midnight χ^2^_15_ = 16.8; p = 0.33). Feeding activity did not cause a significant rise in the vocalisation rate of either call type in the 15 min feeding period (Fig 6).

## Discussion

This current study has shown that the bluefin gurnard have a larger acoustic repertoire than had been previously reported [31]. Two new types of growl types sounds have been identified, which is consistent with at least five other Triglidaeae species also known to growl [8]. This current study also confirmed the presence of the two grunt type sounds, ‘Gu’ and ‘Pons’, previously described for bluefin gurnard [31]. However, for both of these grunt sounds, a slightly shorter pulse duration (0.03–0.14 s versus 0.2 s) and lower peak frequency (95–156 Hz versus 250–300 Hz) were found to those previously described. In addition, a harmonic component with a lower amplitude (5–25 dB lower) compared to the amplitude of the primary peak frequency (Pf1) or fundamental frequency was identified. Harmonics of grunt sounds have not been reported before in other Triglidae species. The occurrence of harmonics is not typical for grunt type sounds but are more common for tonal type swim bladder sounds such as the boatwhistles and hoots produced by Bathracoidids [7]. Nonetheless, harmonics can be generated when grunts are produced in trains as has also been observed in the vocalisation of the three-spined toadfish, *Batrachomoeus trispinosus* [13].The fundamental frequency of grunt sound (129±1.3 Hz and 144±1.4 Hz) could be related to the contraction frequency of the sonic muscle [7]. The growls were aurally distinguishable from grunts by having a lower amplitude, no harmonic, and a relatively broad frequency spectrum.

This current study also revealed that the growls were distinctively produced at night as what appear to be a form of nocturnal vocalisation. It is not clear from this study what activities this nocturnal sound may be associated with. We have discovered that combinations of different sound types within a category (i.e., grunt or growl) could also be combined to form a call that can last up to 11 s. This ability to mix and blend different sound types has also been reported for the Lusitanian toadfish, *Holobatrachus didactylus*, and haddock, *Melanogrammus aeglefinus* [12,22]. The broadly tuned sound for both sound categories had low Q values indicating that the swim bladderis highly damped.

Bluefin gurnard has a relatively large sound repertoire in comparison to those reported for other Triglidae species, such as the European grey gurnard (three types of sound), tub gurnard (two types) and the streaked gurnard (two types) [8,19,21]. The acoustic features of bluefin gurnard vocalisation also showed some marked differences to these other Triglidaes. For example, sound duration for bluefin gurnard (up to 11 s) were typically longer than the above species (up to 3 s) with lower peak frequency (129–215 Hz versus 510, 311, 555 Hz in grey, tub and streaked gurnard, respectively). In other fish families, large vocal repertoires have been reported in a few species such as the mormyrid fish, *Pollimyrus adspersus*, the Lusitanian toadfish and the three-spined toadfish which all produce four or more different types of sound [11–13,39].

In this current study, bluefin gurnard were highly vocal with sounds, particularly grunts, consistently being produced every hour (15.8±6.4 grunts fish^-1^ h^-1^) throughout the 24 h period. Although grunts were more prevalent during dawn compared to day, there was a sustained vocalisation activity during day and night time with no significant difference between the two periods. In contrast, the grey gurnard in captivity showed a different diel pattern of vocal activity, where maximum acoustic activity occurred during the day, minimum activity at night, and intermediate at dawn/dusk (Amorim, 2005). Crepuscular vocalisation peaks are not uncommon amongst marine fishes as had been previously reported for species in several families such as Sciaenidae, Pomacentridae and Batrachoididae [36,40,41].

The bluefin gurnard vocalisations during this study were not found to be associated with feeding activity, unlike those of the grey [42] and streaked gurnard species [21]. For many soniferous fish species, vocalisation is a communication tool that accompanies specific social behaviours [24]. Examples include agonistic calls during competition for territory or food [43], and self-advertising during reproduction [44]. Consequently, vocalisation activity has often been reported to correlate and increase with these behaviours. Asocial vocalization may be associated with distress and bluefin gurnard commonly vocalize after being removed from the water. Distress calls are documented in a number of gurnard species including: Prionotus carolinus; P. evolans; and Black Sea gurnard [8]. Although, the behavioural context of vocalisation for the captive bluefin gurnard could not be determined from this study, the fish were well acclimated to the holding conditions and feeding well indicating that it would be unlikely to be a distress related call. Agonistic behaviour within the group is a possibility, but the observation that call rate was not associated with feeding activity, and the large vocal repertoire and call quantity may indicate an association with reproductive state. Triglidaes are known to be most acoustically active during breeding season [8,45] and the period of this study coincided with the general breeding season during late spring and early summer for the bluefin gurnard in the Hauraki Gulf from where the experimental fish were captured [46]. For other species, the Gulf toadfish, Lusitanian toadfish and the croaker, diel and seasonal variation in their sound production have been found to be associated with behavioural changes during spawning [13,36,44,47].

Understanding the vocalisation repertoire and temporal periodicity of the bluefin gurnard could help in determining the identity of biological sound sources in the local ambient soundscape. The results of this study provided a preliminary insight into the potential contribution of the bluefin gurnard vocalisation to the biological noise of a nearby location (Pakiri Beach) known as gurnard habitat amongst local commercial fishers (pers. obs). A recent study on the ambient noise at this location showed that the proportion of total noise intensity was largest in the frequency band 100–800 Hz [33]. This frequency band encompassed the peak frequency range of bluefin gurnard vocalisation repertoire (Pf1 = 129–215 Hz; Pf2 = 276–290 Hz) suggesting that the bluefin gurnard vocalisation as a viable major source for this sound energy. This would also suggest that the bluefin gurnard as a potential candidate for the non-destructive passive acoustic survey application of their population in the wild. This could help optimise gurnard fishery in New Zealand and is particularly important because although a commercial species, bluefin gurnard is caught as an incidental bycatch of many New Zealand inshore fisheries [48]. In addition, the characteristics, magnitude and temporal pattern of the bluefin gurnard vocalisation show that where they are common, they are likely to be a major contributor to the New Zealand ambient underwater soundscape.
